# How anxiety attributed to COVID-19, disease knowledge, and intention to vaccinate against SARS-CoV-2 viral infection prevail in general public of Saudi Arabia?

**DOI:** 10.3389/fpubh.2023.1078023

**Published:** 2023-02-07

**Authors:** Md. Ashraful Islam, Dhfer Mahdi Alshayban, Atta Abbas Naqvi, Muhammad Bilal Maqsood, Azfar Athar Ishaqui, Muhammad Kashif, Majid Ali, Abdul Haseeb

**Affiliations:** ^1^Department of Pharmacy Practice, College of Clinical Pharmacy, Imam Abdulrahman Bin Faisal University, Dammam, Saudi Arabia; ^2^School of Pharmacy, University of Reading, Reading, United Kingdom; ^3^Eastern Health Cluster, Ministry of Health, Dammam, Saudi Arabia; ^4^Department of Pharmacy, Iqra University, Karachi, Pakistan; ^5^Pharmaceutical Care Department, King Abdulaziz Medical City, Ministry of National Guard Health Affairs, Riyadh, Saudi Arabia; ^6^Department of Basic Sciences, College of Medicine, Sulaiman Al-Rajhi University, Al-Bukayriyah, Saudi Arabia; ^7^Department of Clinical Pharmacy, College of Pharmacy, Umm Al-Qura University, Makkah, Saudi Arabia

**Keywords:** COVID-19, pandemic, COVID-19 vaccine acceptance, vaccine, Saudi Arabia

## Abstract

**Aim:**

The study aimed to document the anxiety attributed to COVID-19, disease knowledge, and intention to vaccinate against the disease in general public. Moreover, the interplay among these three outcomes was also investigated.

**Methods:**

A cross-sectional study was conducted for 2 months in three cities of Dammam Region of Saudi Arabia. The target segment was the adult population of Saudi Arabia. Convenience sampling was used and all adults aged ≥18 were invited to participate. The questionnaire used in the study was available in both Arabic and English languages. It included a demographic section, a section dedicated to vaccination intention and, a section containing coronavirus anxiety scale (CAS). The data analysis was carried out using IBM SPSS version 23. The study was approved by an ethics committee (IRB-2021-05-297).

**Results:**

A total of 542 responses were analyzed. Most respondents had no anxiety attributed to COVID-19 (92.1%), self-reported good knowledge of COVID-19 (79.7%) and intended to administer a vaccine (57.4%). Age groups 18–29 years and 30–45 years, and having a chronic medical condition, were found to be determinants of having COVID-19 anxiety (*p* < 0.05). The variables of self-rated good knowledge of disease, never contracted COVID-19, and incomes of SAR 5,000 (i.e., USD 1333), and SAR 7,500–10,000 (i.e., USD 1999.5–2666), were found to be determinants of having positive intention toward vaccination (*p* < 0.05).

**Conclusion:**

The anxiety due to COVID-19 was present in a few participants. Besides, self-reported knowledge about COVID-19 and intention to administer a vaccine, were positively linked to each other. However, both variables had no effect on COVID-19 anxiety. It is important to review and address the determinants of positive intention to further increase vaccine acceptance rate.

## 1. Introduction

Since March 2020, the world is in the midst of COVID-19 pandemic (1). Since then the virus has been evolving and several new variants of the virus that have high transmission and capability to spread have been reported (2–4). Besides, the daily reporting of new cases and deaths due attributable to COVID-19 was a common occurrence in the news media (5). Such news reports and emotive information propagated a sense of fear and anxiety among the general masses, and it may be linked to anxiety and psychological distress (5, 6). It was also reported that the disease had an effect on social relationships (7).

A study mentions that an individual may respond to fear either rationally or irrationally. A person may respond to the fear of COVID-19 by understanding the threat and preventing the risk (6). However, an irrational response to the fear would be to panic. This may limit the ability of an individual to understand the threat. Moreover, providing more information about the scientifically authentic threat may trigger more panic which would reduce the benefits of the information to the individual (6). As this impact of media information on mental health is observed in this crisis, the WHO has termed this phenomenon as “infodemic.” The term implies that there might be an over-profusion of some reliable and anecdotal information available to the public that makes it difficult to find reliable information at the time of need (5, 8). It was reported that media coverage of the COVID-19 crisis also resulted in stress in the public (5). This sense of fear and panic is detrimental to the mental health of an individual which was already affected due to the containment strategy of lockdowns (9).

During the COVID-19 pandemic, fear of job insecurity was a significant reason for financial anxiety regardless of the employment sector and income among employees in Saudi Arabia (10).

A possible way to reduce occurrence of new COVID-19 infections is through vaccination. This would instigate an immune response in the body and may significantly reduce the likelihood of spread (11). It offers a potential solution to exiting the current crisis (12). A large-scale vaccination drive against COVID-19 is considered as a successful response by the public health authorities in the UK, to address the spread of this viral infection in future (13). A number of vaccine candidates have shown efficacy in clinical trials and have been approved for use in public recently (11, 14, 15).

Saudi health authority approved the first vaccine for the disease as early as December 2020 and started administering vaccines in January 2021. Later in February 2021, the second vaccine was approved for use (16). Initially, large urban centers such as provincial capitals were prioritized for the delivery of vaccines. The vaccine-eligible population was divided into three strata. The first stratum comprised of the healthcare practitioners, geriatrics, military personnel, immune-compromised, obese individuals, and patients with certain chronic diseases. The second stratum comprised of individuals who worked in essential services along with patients with chronic diseases. Both strata had individuals with high risk of COVID-19 and its complications and therefore, were prioritized to receive a vaccine by July 2021. The general public was in the third stratum and was prioritized to be vaccinated by September 2021 (17).

However, how anxious the general public is due to COVID-19 infection, their level of awareness and the intention to vaccinate against the viral infection needs to be seen. In addition, it is worthwhile documenting how these three factors affect each other and the overall vaccination intention.

## 2. Methods

### 2.1. Objective

The study aimed to report the anxiety attributed to COVID-19, self-rated disease knowledge, and intention to vaccinate against the disease among general public. Moreover, the interplay among these three outcomes was also investigated.

### 2.2. Design, duration, and venue

This study was designed as a cross-sectional survey and was conducted for 2 months, i.e., September–October 2021 in three cities of the Dammam Region of Saudi Arabia.

### 2.3. Target population and eligibility

The target segment was the adult population. Adult male and female participants who aged 18 and above and eligible for a vaccine, were invited to participate in this study. Incomplete responses were excluded from study.

### 2.4. Sampling and data collection

The data was collected using a convenient sampling method. Participants who could be conveniently approached were contacted. The mode of survey was physical as well as online. Both forms were available as per the convenience of participants. The online survey was conducted using an electronic tablet, while the hardcopies were handed to participants and later collected. The venues selected were mostly open public spaces such as public parks and open areas of shopping malls. The data was collected once per participant and there was no follow-up.

### 2.5. Sample size calculation

The sample size was calculated using an online sample size calculator. Dammam Region consists of several cities. Our study included cities namely Dammam, Dhahran, and Khobar. According to available estimates at the time of this writing, the population of Dammam city was 7,68,602 while Dhahran and Khobar had a population of 99,540 and 1,65,799 respectively (18). Since there was no description of numbers related to the vaccine eligible population, the sum of all three figures, i.e., population of 1,033,941 individuals, was considered the total population of these cities and thus, our target population.

The sample size was calculated using a margin of error of 5% and a confidence interval of 97.5%. From the initial calculation, the number of samples obtained was 503 (19). Later, the sample size was adjusted for non-response/missing error rate which was considered at 20%. Finally, the required sample size turned to 629.

### 2.6. Research questionnaire

The research questionnaire consisted of a demographic section, a section dedicated to vaccination intention and, a section containing Coronavirus Anxiety Scale (CAS) (20). The demographic section contained questions related to the age, sex, nationality, education level, marital status, occupation, residence, and income. The second section contained items related to the participants' medical history, any exposure to COVID-19, self-rated knowledge about COVID-19, level of compliance to recommendations aimed at preventing COVID-19 spread, and vaccination intention. The third section was CAS itself, a validated scale to measure anxiety related to the disease. The scale contained 4 items related to the COVID-19 anxiety on a person's daily life in last 14 days. The scale was Likert-format, and each item had 5 possible options. Each options awarded a score. A cumulative score of ≥ 9 indicated COVID-19 anxiety (20).

A formal permission was obtained from the developer of the scale through email. The questionnaire was formulated in native Arabic and English languages. The CAS scale was already available in both English and Arabic languages at the time of study. The survey was piloted in few participants before actual study.

### 2.7. Data management

Data were checked for incomplete and missing responses. At the beginning 558 responses were received and 71 samples of incomplete responses were excluded from the data set. Of 23 partially incomplete responses, 16 were excluded due to untreatable nature, whereas 7 (seven) responses were treated using the “last observation carried forward” statistical method. Finally, a total of 542 complete responses were analyzed. The potential sources of bias considered during this study were selection bias due to the convenient sampling, and information bias attributed to the self-reporting format of this study. The study outcomes were the intention to vaccinate and COVID-19 anxiety. A secondary outcome considered was COVID-19 self-reported knowledge.

### 2.8. Data analysis

The data were coded and entered for analysis in IBM SPSS version 23. The demographic data was reported using sample counts (N) and percentages (%) for descriptive data. Simple and multiple logistic regression methods were used to report the determinants of the outcomes. Only the significant variables were included in the multiple regression model. The significance of those variables was determined by simple regression analysis and necessary model fitness parameters were checked by required statistics that are mentioned in footnotes of **Tables 4**–**6**.

### 2.9. Consent and ethical clearance

The participants were briefed about the study and their consent was sought. The participation was voluntary. An electronic informed consent was designed and was shown at the front page of the online survey, and it was also available in hardcopy. The participants in the online survey could only access the survey if they consent to participate while those who preferred the hardcopy were asked to provide their consent before accessing the survey. The nature of consent was implied, i.e., participants were not required to provide their personal identifiable details. The study was approved by the Institutional Review Board of Imam Abdulrahman Bin Faisal University, Dammam, Saudi Arabia (IRB -2021-05-297).

## 3. Results

A total of 542 responses were analyzed.

### 3.1. Background characteristics

Most respondents were female (*N* = 352, 64.9%) and aged between 18 and 29 years (*N* = 306, 56.5%). Most of them were Saudi nationals (*N* = 513, 94.6%), students (*N* = 219, 40.4%), resided in urban area (*N* = 457, 84.3%) and had postgraduate qualification (*N* = 230, 42.4%). Slightly less than half (*N* = 237, 43.7%) had a monthly family income above Saudi Arabian Riyal (SAR) 10,000, i.e., > United States Dollar (USD) 2666, (Table 1).

**Table 1:** Background characteristics of participants (*N* = 542).

**Characteristics**	**Frequency (*N*)**	**Percent (%)**
Age		
18–29	306	56.5
30–45	113	20.8
46–64	107	19.7
65 and more	16	3
Sex		
Male	190	35.1
Female	352	64.9
Nationality		
Non-Saudi	29	5.4
Saudi	513	94.6
Education level		
Primary education	2	0.4
Secondary education	12	2.2
Higher secondary education	90	16.6
Undergraduate	208	38.4
Postgraduate	230	42.4
Marital status		
Single	271	50
Married	271	50
Occupation		
Employed or self-employed	138	25.5
Unemployed or retired	79	14.6
Student	219	40.4
Homemaker	106	19.6
Income^*^		
SAR 5,000 (USD 1,333)	134	24.7
SAR 5,000–7,500 (USD 1,333–1,999.5)	82	15.1
SAR 7,500–10,000 (USD 1,999.5–2,666)	89	16.4
Above SAR 10,000 (>USD 2,666)	237	43.7
Residence		
Urban	457	84.3
Rural	85	15.7

^*^1 USD equals SAR 3.75.

### 3.2. Medical information of participants

Most participants did not have any chronic illnesses (*N* = 449, 82.8%) and mental illnesses (*N* = 492, 90.8%). More than half of participants rated their knowledge of COVID-19 as good (*N* = 304, 56.1%). The majority did not suffer from COVID-19 (*N* = 458, 84.5%) however, had COVID-19 patients in their family (*N* = 469, 86.5%). Most of the participants never missed a doctor recommended vaccine (*N* = 508, 93.7%) and intended to vaccinate against COVID-19 (*N* = 311, 57.4%) (Table 2).

**Table 2:** Medical information of participants (*N* = 542).

**Medical information**	***N***	**%**
Presence of any chronic physical health condition (e.g., diabetes, arthritis, cardiac diseases, etc.)		
Yes	93	17.2
No	449	82.8
Presence of any long-term mental health condition (e.g., depression, stress, anxiety, etc.)		
Yes	50	9.2
No	492	90.8
Self–rated COVID-19 knowledge		
No knowledge	4	0.7
Poor knowledge	17	3.1
Little knowledge	89	16.4
Good knowledge	304	56.1
Excellent knowledge	128	23.6
Suffered from COVID-19?		
Yes	84	15.5
No	458	84.5
Anyone in family/friends/relatives suffered from COVID-19?		
Yes	469	86.5
No	73	13.5
Ever refused or elected to forego a doctor recommended vaccine?		
Yes	34	6.3
No	508	93.7
How likely to do you think you are to get a COVID-19 vaccine when one is approved?		
Intend to vaccinate against COVID-19	311	57.4
Undecided on COVID-19 vaccination	158	29.2
Do not intend to vaccinate against COVID-19	73	13.5

### 3.3. Study outcomes

**Figure 1:**
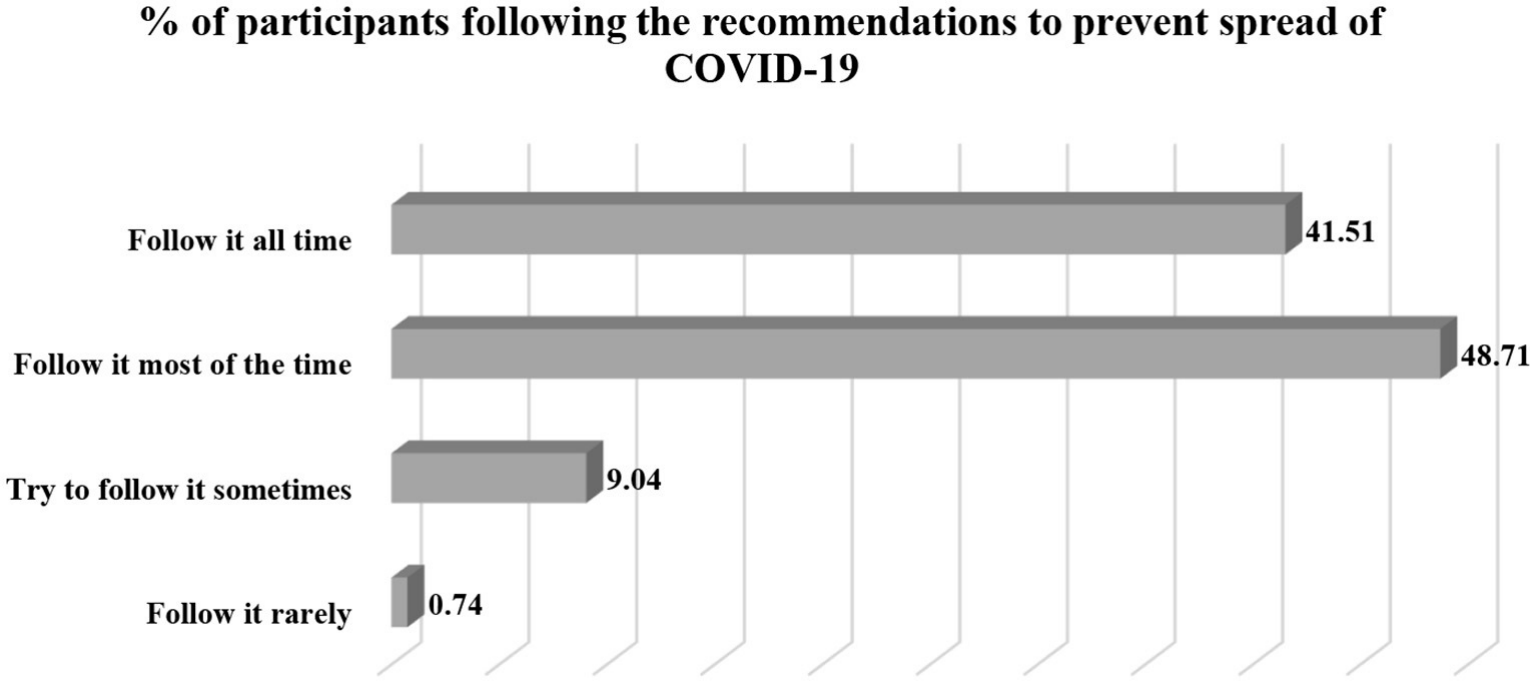
Percentage distribution of participants regarding compliance to COVID-19 prevention guidelines.

Slightly less than half of the participants mentioned that they follow the recommendations from authorities regarding COVID-19 prevention at most times (Figure 1).

The majority reported an acceptable level of knowledge of COVID-19 viral infection (*N* = 432, 79.7%). More than half of the participants had positive intent regarding COVID-19 vaccination (*N* = 311, 57.4%). Most of them had no anxiety attributed to COVID-19 (*N* = 499, 92.1%). The reliability of the Coronavirus Anxiety Scale (CAS) was 0.845, i.e., Cronbach's alpha value (Table 3).

**Table 3:** Characteristics of outcome variables (*N* = 542).

**Outcomes**	**Frequency (*N*)**	**Percent (%)**
Acceptable level of knowledge for COVID−19 infection		
No	110	20.3
Yes	432	79.7
Intention to vaccinate against COVID-19		
Not positive	231	42.6
Positive	311	57.4
COVID-19 anxiety		
Absent	499	92.1
Present	43	7.9

The model for COVID-19 anxiety highlighted those individuals in age groups 18–29 and 30–45 years were more likely to have COVID-19 anxiety, i.e., (AOR 5.33) and (AOR 6.48), respectively, compared to individuals aged 46 years and above, when adjusted for other demographics (*p* < 0.05). Besides, individuals who mentioned having any long-term mental health condition were roughly three times more likely to have COVID-19 anxiety (AOR 2.66) compared to those without any mental illness when other demographics are considered (*p* < 0.05). Some of the variables were significant determinants of COVID-19 anxiety alone and became non-significant when adjusted for all different demographics (Table 4).

**Table 4:** Determinants of COVID-19 anxiety.

**Characteristics**	**OR (95% CI of OR)**	**AOR (95% CI of OR)**
Age in years		
46 and above (R)	— — —	— — —
18–29	6.09 (1.43, 25.98)^*^	5.33 (1.04, 27.25)^*^
30–45	7.87 (1.73, 35.68)^**^	6.48 (1.30, 32.25)^*^
Gender		
Female (R)	— — —	— — —
Male	0.62 (0.30, 1.25)	— — —
Nationality		
Saudi (R)	— — —	— — —
Non-Saudi	1.36 (0.39, 4.71)	— — —
Education level		
Postgraduate (R)	— — —	— — —
Up to higher secondary level of education	1.12 (0.46, 2.69)	— — —
Undergraduate	1.35 (0.67, 2.69)	— — —
Marital status		
Married (R)	— — —	— — —
Single	1.59 (0.84, 2.99)	— — —
Occupation		
Employed or self-employed (R)	— — —	— — —
Unemployed or retired	1.17 (0.32, 4.29)	1.81 (0.43, 7.72)
Student	2.33 (0.92, 5.94)	1.93 (0.62, 6.04)
Homemaker	2.81 (1.02, 7.75)^*^	2.56 (0.84, 7.87)
Income		
Above SAR 10,000 (R)	— — —	— — —
SAR 5,000	2.91 (1.36, 6.25)^**^	2.08 (0.93, 4.65)
Between SAR 5,000 and 7,500	1.22 (0.42, 3.57)	1.18 (0.39, 3.53)
Between SAR 7,500 and 10,000	1.85 (0.73, 4.69)	1.81 (0.69, 4.76)
Residence		
Urban (R)	— — —	— — —
Rural	1.05 (0.45, 2.44)	— — —
Having any long-term physical health condition (e.g., diabetes, arthritis, cardiac diseases, etc.)?		
No (R)	— — —	— — —
Yes	1.52 (0.72, 3.20)	— — —
Having any long-term mental health condition (e.g., depression, stress, anxiety, etc.)?		
No (R)	— — —	— — —
Yes	2.96 (1.33, 6.59)^**^	2.66 (1.14, 6.23)^*^
Self-rated knowledge of COVID-19		
Good knowledge (R)	— — —	— — —
Poor knowledge	1.04 (0.48, 2.24)	— — —
Previously contracted COVID-19		
No (R)	— — —	— — —
Yes	1.27 (0.59, 2.85)	— — —
Any family member/friends/relatives suffered from COVID-19		
No (R)	— — —	— — —
Yes	2.18 (0.66, 7.22)	— — —
Ever refused or elected to forego a doctor recommended vaccine for self or a dependent (e.g., child)?		
No (R)	— — —	— — —
Yes	2.73 (1.06, 7.01)^*^	3.28 (1.18, 9.12)
Having positive intention to administer COVID-19 vaccine		
Yes (R)	— — —	— — —
No	1.07 (0.57, 2.01)	— — —

R, reference case; OR, Odds ratio; CI, Confidence Interval; AOR, Adjusted odds ratio.

^*^P < 0.05 and ^**^P < 0.01.

Multiple logistic regression, “Enter” method was applied; Multicollinearity was checked and not found; Hosmer-Lemeshow test, (χ2 = 4.33, p = 0.741); Pearson chi-square and Significance for Model (χ2 = 29.86 and p < 0.01) and Classification table, (overall correctly classified percentage = 92.1) were applied to check the model fitness. Cox and Snell R Square = 0.054; Nagelkerke R Square = 0.126.

The model for a positive intention toward COVID-19 vaccination reported that compared to the females, the male respondents had a higher likelihood of positive intention (AOR 2.27) after adjusting for all demographic characteristics (*p* < 0.001). Besides, those who were single (AOR 1.77) had a higher likelihood of positive intention (*p* < 0.01). Further, compared to those individuals having a monthly income above SAR 10,000, those with an income of SAR 5,000 (AOR 0.59) and SAR 7,500–10,000 (AOR 0.57), had negative intention when adjusted for other demographics (*p* < 0.05). Individuals who rated their knowledge of COVID-19 as good were more likely to vaccinate (AOR 1.78), while those who never contracted the disease showed positive intention (1.74) toward vaccination when adjusted for all other demographics (*p* < 0.05). Some of the variables were significant determinants of positive intention alone and become non-significant when adjusted for all other demographics (Table 5).

**Table 5:** Determinants of positive intention toward COVID-19 vaccination.

**Predictors**	**OR (95% CI of OR)**	**AOR (95% CI** **of OR)**
Age		
Age 30 years and more (R)	— — —	— — —
Age < 30 years	1.56 (1.10, 2.19)^*^	0.87 (0.47, 1.59)
Gender		
Female (R)	— — —	— — —
Male	2.37 (1.63, 3.44)^***^	2.27 (1.45, 3.55)^***^
Nationality		
Non-Saudi (R)	— — —	— — —
Saudi	3.18 (1.42, 7.12)^**^	3.84 (1.60, 9.24)^**^
Education level		
Up to higher secondary level of education (R)	— — —	— — —
Undergraduate and postgraduate level of education	1.84 (1.19, 2.83)^**^	1.46 (0.86, 2.49)
Marital status		
Married (R)	— — —	— — —
Single	1.93 (1.36, 2.72)^***^	1.77 (1.09, 3.19)^*^
Occupation		
Homemaker (R)	— — —	— — —
Employed or self-employed	1.37 (0.82, 2.28)	0.85 (0.47, 1.56)
Unemployed or retired	2.16 (1.19, 3.93)^*^	1.02 (0.50, 2.07)
Student	2.32 (1.44, 3.72)^**^	0.88 (0.40, 1.95)
Income		
Above SAR 10,000 (R)	— — —	— — —
SAR 5,000	0.57 (0.37, 0.87)^*^	0.59 (0.36, 0.98)^*^
Between SAR 5,000 and 7,500	0.52 (0.31, 0.86)^*^	0.62 (0.35, 1.09)
Between SAR 7,500 and 10,000	0.51 (0.31, 0.83)^**^	0.57 (0.33, 0.98)^*^
Residence		
Rural (R)	— — —	— — —
Urban	1.64 (1.03, 2.61)^*^	1.31 (0.78, 2.19)
Having any long-term physical health condition (e.g., diabetes, arthritis, cardiac diseases, etc.)?		
Yes (R)	— — —	— — —
No	1.26 (0.80, 1.97)	— — —
Having any long-term mental health condition (e.g., depression, stress, anxiety, etc.)?		
Yes (R)	— — —	— — —
No	1.27 (0.62, 2.04)	— — —
Self-rated knowledge of COVID-19		
Poor knowledge (R)	— — —	— — —
Good knowledge	1.92 (1.26, 2.93)^**^	1.78 (1.12, 2.84)^*^
Previously contracted COVID-19		
Yes (R)	— — —	— — —
No	1.69 (1.06, 2.69)^*^	1.74 (1.04, 2.91)^*^
Any family member/friends/relatives suffered from COVID-19		
No (R)	— — —	— — —
Yes	1.13 (0.69, 1.85)	— — —
Ever refused or elected to forego a doctor recommended vaccine for self or a dependent (e.g., child)?		
No (R)	— — —	— — —
Yes	0.09 (0.030, 0.25)^***^	0.08 (0.03, 0.23)
Having COVID-19 disease anxiety		
Yes (R)	— — —	— — —
No	1.07 (0.57, 2.01)	— — —

R, reference case; OR, Odds ratio; CI, Confidence Interval; AOR, Adjusted odds ratio.

^*^P < 0.05, ^**^P < 0.01, and ^***^P < 0.001.

Multiple logistic regression, “Enter” method was applied; Multicollinearity was checked and not found; Hosmer-Lemeshow test, (χ2 = 10.56, p = 0.228); Pearson chi-square and Significance for Model (χ2 = 98.22 and p < 0.001) and Classification table, (overall correctly classified percentage = 69.2) were applied to check the model fitness. Cox and Snell R Square = 0.166; Nagelkerke R Square = 0.223.

The model for self-rated good knowledge of COVID-19 disease revealed that compared to individuals who lived in rural localities, individuals living in urban areas were more likely to rate their knowledge as good (AOR 1.71) when other demographics were considered (*p* < 0.05). Besides, individuals who did not have any long-term mental health condition were three times more likely to rate their knowledge of COVID-19 as good (AOR 2.74) when adjusting other demographics (*p* < 0.01). Moreover, individuals who intended to receive COVID-19 vaccine were more likely to rate their knowledge of the disease as good (AOR 1.75) when other variables were considered (*p* < 0.05). Some of the variables were significant determinants of positive intention alone and become non-significant when adjusted for all other demographics (Table 6).

**Table 6:** Determinants of good knowledge of COVID-19 disease.

**Predictors**	**OR (95% CI of OR)**	**AOR (95% CI of OR)**
Age		
Age 30 years and more (R)	— — —	— — —
Age < 30 years	1.75 (1.15, 2.66)^**^	1.36 (0.72, 2.59)
Gender		
Female (R)	— — —	— — —
Male	1.03 (0.66, 1.60)	— — —
Nationality		
Non-Saudi (R)	— — —	— — —
Saudi	0.97 (0.39, 2.45)	— — —
Education level		
Up to higher secondary level of education (R)	— — —	— — —
Undergraduate and postgraduate level of education	1.93 (1.19, 3.14)^**^	1.48 (0.86, 2.56)
Marital status		
Married (R)	— — —	— — —
Single	1.59 (1.04, 2.42)^*^	1.17 (0.63, 2.13)
Occupation		
Others (unemployed or retired, student and homemaker) (R)	— — —	— — —
Employed or self-employed	1.13 (0.69, 1.85)	— — —
Income		
SAR 5,000 (R)	— — —	— — —
Between SAR 5,000 and 7,500	0.75 (0.39, 1.43)	— — —
Between SAR 7,500 and 10,000	1.62 (0.79, 3.31)	— — —
Above SAR 10,000	1.15 (0.68, 1.93)	— — —
Residence		
Rural (R)	— — —	— — —
Urban	1.82 (1.08, 3.07)^*^	1.71 (1.01, 2.93)^*^
Having any long-term physical health condition (e.g., diabetes, arthritis, cardiac diseases, etc.)?		
Yes (R)	— — —	— — —
No	1.49 (0.87, 2.47)	— — —
Having any long-term mental health condition (e.g., depression, stress, anxiety, etc.)?		
Yes (R)	— — —	— — —
No	2.45 (1.31, 4.55)^**^	2.74 (1.44, 5.20)^**^
Previously contracted COVID – 19		
Yes (R)	— — —	— — —
No	0.99 (0.56, 1.78)	— — —
Any family member/friends/relatives suffered from COVID-19		
No (R)	— — —	— — —
Yes	1.12 (0.62, 2.04)	— — —
Ever refused or elected to forego a doctor recommended vaccine for self or a dependent (e.g., child)?		
No (R)	— — —	— — —
Yes	0.69 (0.31, 1.52)	— — —
Having COVID-19 disease anxiety		
Yes (R)	— — —	— — —
No	1.04 (0.49, 2.25)	— — —
Having positive intention to administer COVID-19 vaccine		
No (R)	— — —	— — —
Yes	1.92 (1.26, 2.93)^**^	1.75 (1.13, 2.71)^*^

R, reference case; OR, Odds ratio; CI, Confidence Interval; AOR, Adjusted odds ratio.

^*^P < 0.05, and ^**^P < 0.01.

Multiple logistic regression, “Enter” method was applied; Multicollinearity was checked and not found; Hosmer-Lemeshow test, (χ2 = 3.430, p = 0.753); Pearson chi-square and Significance for Model (χ2 = 28.95 and p < 0.001) and Classification table, (overall correctly classified percentage = 79.9) were applied to check the model fitness. Cox and Snell R Square = 0.052; Nagelkerke R Square = 0.082.

## 4. Discussion

The interplay of COVID-19 disease anxiety, self-rated disease knowledge, and vaccine intention has not been investigated so far among general public in Saudi Arabia. Although, studies have documented knowledge, vaccination acceptance, and fear of the disease as standalone concepts (21–25). However, how these entities affect each other remains undocumented. The findings of this study highlighted that the COVID-19 anxiety appeared independent of the disease knowledge and vaccine acceptance. However, the latter two appeared to influence each other as both were reported as significant determinants for each other. There is logic in this finding as knowledge of COVID-19 has the potential to improve the understanding about vaccines for the same. The COVID-19 knowledge had a positive impact on vaccination intention. However, it does not affect the COVID-19 anxiety which appeared to be influenced by the mental health of respondent.

### 4.1. Self-rated disease knowledge

Our findings highlight that the self-rated knowledge of COVID-19 among most participants was at an acceptable level. This confirmed the findings of previous studies which reported that public in Saudi Arabia is quite aware of the infection (26–28). However, one of the study reported that it may be higher among individuals with higher education and income (28). In our study, most participants had higher education and income. To this end, this study adds to the existing body of literature by carrying out the analysis to report the determinants of self-rated good knowledge of COVID-19. The residence may impact an individual's life. People living in urban areas may have more opportunities for obtaining education and finding better work as most educational institutes and work related opportunities are usually concentrated in urban areas. Our findings reveal that individuals who resided in urban localities were more likely to rate their knowledge as good compared to those living in rural areas. Besides, individuals with a higher degree such as bachelor or a postgraduate qualification were more likely to rate their knowledge as good compared to others. However, the variable of higher education was only a significant determinant when other demographics were not considered. Further, our data could not establish the aspect of income as a significant determinant of self-rated good knowledge of the infection. In another study, it was reported that older participants were more knowledgeable (26). However, in our study we found that participants aged 30 years or younger were more likely to rate their knowledge as good. Though, the variable of age was only significant when other variables were not considered.

Our findings highlight that individuals who did not suffer from any long-term mental health condition were more likely to rate their knowledge of COVID-19 as good. This aspect of viewing mental health issues alongside knowledge of disease was not reported from this population. This finding also raises the question of evaluating the impact of mental health on disease knowledge. In this context, an interesting finding was reported by Al Dhaheri et al. (29) that the disease had some level of psychological impact. It was also observed in our study that the individuals who intended to receive a vaccine for the disease were roughly two times more likely to rate their knowledge as good.

### 4.2. Vaccine acceptance

In regards to the intention to receive a vaccine for COVID-19, more than half of the target segment, i.e., 57.4% had a positive intention to receive a vaccine. This was similar to the results reported by previous studies (30, 31). In addition, the data highlights that the male respondents and those who never contracted the disease before, had a higher likelihood of positive intention as compared to female respondents and individuals with a history of COVID-19 infection, respectively. Being male and having no history of COVID-19 infection were previously reported as predictors of positive intention in this population (31). In addition, our data highlighted that individuals who rated their knowledge of COVID-19 as good were more likely to vaccinate. A possible explanation for this occurrence could be that better understanding about the disease may prompt an individual to seek a positive change, i.e., treatment. Hence, patients who rated their knowledge as good understood the disease and its treatment. Therefore, they indicated their acceptance for vaccination.

### 4.3. COVID-19 anxiety

It was also observed that most respondents had no anxiety owing to the viral infection. Individuals with long-term mental illnesses appeared prone to having COVID-19 anxiety. This was in line with results of a previous study where it was reported that the disease had a psychological impact (29). Therefore, it is likely that respondents in our study who suffered from mental illness were more anxious. Our study reported that younger individuals were more likely to have anxiety as compared to the older ones. To this end, a previous study reported a high level of anxiety in young adults between 18 and 29 years during COVID-19 resultant lockdown in Saudi Arabia (32). Thus, our findings highlight the presence of continued anxiety in this age group. This age group usually contains individuals who are either involved in their studies or are working. COVID-19 has caused disruptions in education and work sectors such as switching to online education (33). It has also resulted in switching to work from home (34). This coupled with new outbreaks and emergence of new variants of the virus may continuously bother such individuals. It is extremely important to address the mental issues pertaining to individuals in this age group.

### 4.4. Study limitations

The study has some limitations, due to the convenient nature of data collection, there may be a selection bias as the researchers gather data from individuals who could be easily approached. Although the study gathered data from a large number of participants with reasonable demographic diversity, the findings should be interpreted with caution. This also affects the generalizability of the findings. However, since it was carried out in several sites at different times of day. This would have offered a considerable level of random data collection within the convenient realm. Therefore, it is expected that the findings may be limited to local settings and may not be generalized.

Secondly, there may an element of information bias attributed to the self-reporting format of this study particularly with answers related to the COVID-19 disease anxiety, disease knowledge, compliance to social distancing protocols provided by the health authority. We believe that there may be a slight overestimation by respondents as they may have tended to select a socially acceptable response. Although, they were informed that the data was collected without any personal identifier, and they cannot be identified once the response has been submitted. Besides, it was explicitly requested to provide an honest response for this exact purpose. Moreover, some of the findings of this study were similar to the ones reported previously. Lastly, study gathered data from a small number of non-Saudi respondents and therefore, all comparisons made based on nationality must be interpreted with caution.

## 5. Conclusion

The findings of this study highlight that COVID-19 knowledge had a positive impact on vaccination intention however, it does not affect COVID-19 anxiety. It is recommended to initiate more disease awareness campaigns in small towns and rural communities as individuals hailing from those localities had low knowledge and subsequently less positive intent. Secondly, mental conditioning sessions may be required for students and employees at universities and workplaces respectively as these groups had a higher likelihood of COVID-19 anxiety.

## Data availability statement

The raw data supporting the conclusions of this article will be made available by the authors, without undue reservation.

## Ethics statement

The studies involving human participants were reviewed and approved by Institutional Review Board of Imam Abdulrahman Bin Faisal University, Dammam, Saudi Arabia (IRB−2021-05-297). The patients/participants provided their written informed consent to participate in this study.

## Author contributions

MI: conceptualization, methodology, questionnaire design, ethical clearance, formal analysis, writing–original draft, correspondence, revision of manuscript, and preparing response to reviewers' comments. DA: methodology, questionnaire design, writing–original draft, revision of manuscript, preparing response to reviewers' comments, and supervision of process. AN: conceptualization, methodology, questionnaire design, writing–original draft, revision of manuscript, and preparing response to reviewers' comments. MM and AI: methodology, questionnaire design, data collection, revision of manuscript, and preparing response to reviewers' comments. MK: questionnaire design, data collection, and revision of manuscript. MA: methodology, questionnaire design, revision of manuscript, and preparing response to reviewers' comments. AH: methodology, questionnaire design, revision of manuscript, funding acknowledgment, and supervision of process.
