# Supplementary material for: How anxiety attributed to COVID-19, disease knowledge, and intention to vaccinate against SARS-CoV-2 viral infection prevail in general public of Saudi Arabia?
Source: Front Public Health. 2023 Feb 7;11:1078023. doi: 10.3389/fpubh.2023.1078023 (PMC9941530; doi:10.3389/fpubh.2023.1078023)
Supplement: Supplementary file 1 [file Data_Sheet_1.DOCX]

**استمارة الموافقة للمشاركين**

| **الجزء الأول : معلومات المرضى** |
| --- |
| شكرًا لأخذك من وقتك للمشاركة باستبياننا! نريد أن نعرف مستوى القلق من فيروس كورونا ونية أخذ لقاح كوفيد-19 بالمستقبل القريب. لهذا السبب، نحن قائمون على دراسة لتوثيق هذه الممارسة بالمجتمع السعودي. هذه الدراسة تدعوا قرابة 600 شخص لتعبأة هذا الاستبيان بخصوص ما ذكر. هذه العملية ستأخذ قرابة 10 – 15 دقيقة. نرجوا الانتباه أن مشاركتكم اختيارية، وليس لديكم أي التزام لمشاركة الدراسة. لم يتم سؤالكم عن أي معلومات شخصية قد تمكن معرفتكم. نسألكم بمساعدتنا أن نتعلم أكثر بخصوص مستوى القلق من فيروس كورونا ونية أخذ لقاح كورونا بالمستقبل القريب. ندعوكم للمشاركة بهذا المشروع البحثي. إن كنت موافق، سيطلب منك:   1. تعبأة استبيان عبر الانترنت، أو استبيان يعبّأ يدويًآ، متوفر بالإنجليزية والعربية. 2. تجيب عن أسئلة تخص نفسك، أو بالإمكان قراءتها لك وبإمكانك الإجابة شفويًا للإجابة الذي ترغب بكتابتها.   المعلومات التي ستسجل سرية، ولا أحد سوانا سيكون لديه إمكانية وصول لاستبيانك. لن يكون هناك عوائد مباشرة لك، ولكن مشاركتك ستساعدنا على اكتشاف المزيد بخصوص مستوى القلق من فيروس كورونا ونية أخذ لقاح كوفيد-19 بمجتمعك. لو كان لديك أي سؤال، بإمكانك سؤال ممثلنا.   \| **الجزء الثاني : اثبات الموافقة** \| \| --- \| \| لقد تم دعوتي للمشاركة بهذا البحث مستوى القلق من فيروس كورونا ونية أخذ لقاح كوفيد-19 بالمستقبل القريب. لقد تم ابلاغي أنه لن يكون لي مخاطر بالمشاركة. أنا مدرك أنه لن يكون عائد شخصي لي. لقد قرأت المعلومات بالأعلى، أو تم قراءتها لي. كانت لدي الفرصة لأن أسأل الأسئلة بخوصها وأي سؤال سألته تمت الإجابة عنه بشكل مرضي. أوافق بشكل اختياري لأكون مشارك بهذه الدراسة وأتفهم أنه لدي الحق للانسحاب منها بأي وقت بدون أن تؤثر بأي طريقة على رعايتي الصحية. \| |

- أوافق بالمشاركة بهذا الاستبيان
- لا أوافق بالمشاركة بهذا الاستبيان

**القسم الأول : المعلومات السكانية**

1. **العمر بالسنوات**

- ١٨ - ٢٩
- ٣٠ - ٤٥
- ٤٦ - ٦٤
- ٦٥ وفوق

1. **الجنس**

- ذكر
- أنثى

1. **مستوى التعليم**

- تعليم ابتدائي
- تعليم متوسط
- تعليم ثانوي
- جامعي بقيد الدراسة
- جامعي متخرج من الدراسة

1. **الحالة الاجتماعية**

- أعزب
- متزوج

1. **المهنة**

- موظف او عمل مستقل
- غير متوظف او متقاعد
- طالب
- رب منزل

1. **دخل العائلة الشهري**

- أقل من ٥٠٠٠ ريال
- من ٥٠٠٠ إلى ٧٥٠٠ ريال
- من ٧٥٠٠ ريال إلى ١٠٠٠٠ ريال
- أكثر من ١٠٠٠٠ ريال

1. **مكان السكن**

- المدن
- القرى

1. **هل تعاني من أي من الأمراض الجسدية المزمنة ( مثل السكري، التهاب المفاصل، الأمراض القلبية كالضغط الخ...) ؟**

- نعم
- لا

1. **هل تعاني من أي من الأمراض النفسية المزمنة ( مثل الاكتئاب، اضطراب القلق، إجهاد، الخ.. ) ؟**

- نعم
- لا

**القسم الثاني : معلومات متعلقة بفيروس كورونا٥**

1. **كم كيف تقيم معرفتك عن كوفيد – ١٩ ؟ من مقياس ١ إلى ٥**

- ١ = عدم معرفة
- ٢ = معرفة ضعيفة
- ٣ = معرفة قليلة
- ٤ = معرفة جيدة
- ٥ = معرفة ممتازة

1. **هل تتبع التوصيات والاحترازات لمنع انتشار كوفيد – ١٩ ؟**

- أتبعها بشكل نادر
- أحاول أن أتبعها أحيانًا
- أتبعها معظم الوقت
- أتبعها كل الوقت

1. **هل أًصبت بكوفيد --19 ؟**

- نعم
- لا

1. **هل هناك أحد من عائلتك/أصدقاؤك/ أقاربك أصيبوا بكوفيد– 19؟**

- نعم
- لا

1. **هل سبق ورفضت أو ساهمت برفض توصيات طبيب لأخذ لقاح لك أو لأحد أنت مسؤول عنه ( مثل طفلك ) ؟**

- نعم
- لا

1. **ما احتمالية أخذك لقاح كوفيد – 19 ؟**

- أنوي أن أخذ لقاح كوفيد – 19
- لم أحسم رأيي عن لقاح كوفيد – 19
- لا أنوي أن آخذ لقاح كوفيد – 19
