# Supplementary material for: How anxiety attributed to COVID-19, disease knowledge, and intention to vaccinate against SARS-CoV-2 viral infection prevail in general public of Saudi Arabia?
Source: Front Public Health. 2023 Feb 7;11:1078023. doi: 10.3389/fpubh.2023.1078023 (PMC9941530; doi:10.3389/fpubh.2023.1078023)
Supplement: Supplementary file 2 [file Data_Sheet_2.DOCX]

**INFORMED CONSENT FORM FOR PARTICIPANTS**

| **PART I: PATIENT INFORMATION** |
| --- |
| Thank you for giving your time to our survey! We want to know about public attitude towards vaccination against COVID – 19 disease in near future. For this purpose, we are conducting a study to document this practice in Saudi public. This study will invite around 600 people to fill in a survey regarding the same. This process will hardly take 10 – 15 mins. Please note that your participation is voluntary, and you have no obligation to join the study. You will not be asked for any personal information that could identify you and your response will be anonymous. Additionally, you may withdraw your participation at any time. We are asking you to help us learn more about the attitude towards a possible COVID – 19 vaccine in near future. We are inviting you to take part in this research project. If you accept, you will be asked to:  1. fill out an online survey, or hand filled survey, available in English and Arabic.  2. You may answer the questionnaire yourself, OR it can be read to you, and you can say out loud the answer you want us to write down.  The information recorded is confidential, and no one else except us will have access to your survey. There will be no direct benefit to you, but your participation is likely to help us find out more about the attitude towards a possible COVID – 19 vaccination in your community. If you have any questions, you can ask our representative.   \| **PART II: CERTIFICATE OF CONSENT** \| \| --- \| \| I have been invited to participate in research about my knowledge of medication use and safety and adverse drug reactions reporting and pharmacovigilance. I have been informed that there are no risks in participation. I am aware that there may be no benefit to me personally. I have read the above information, or it has been read to me. I have had the opportunity to ask questions about it and any questions. I have asked, have been answered to my satisfaction. I consent voluntarily to be a participant in this study and understand that I have the right to withdraw from the study at any time without in any way affecting my medical care. \| |

- I agree to participate in the survey
- I do not agree to participate in the survey

**Section 1: Demographic information**

| **1. Age in years** |
| --- |
| - 18 – 29 - 30 – 45 - 50 – 64 - 65 and above |
| 1. **2. Gender** |
| - Male - Female |
| 1. **3. Level of education** |
| - Primary education - Secondary education - Higher secondary education - Undergraduate - Postgraduate |
| **4. Marital status** |
| - Single - Married |
| 1. **5. Occupation** |
| - Employed or self-employed - Unemployed or retired - Student - Homemaker |
| 1. **6. Monthly family income** |
| - Less than SAR 5000 - SAR 5,000 – 7,500 - SAR 7,500 – 10,000 - Above SAR 10,000 |
| 1. **7. Residence** |
| - Urban - Rural |
| 1. **8. Do you have any long-term physical health condition (for example diabetes, arthritis, cardiac diseases, etc.)?** |
| - Yes - No |
| 1. **9. Do you have any long-term mental health condition (for example depression, anxiety, stress, etc.)?** |
| - Yes - No |

**Section 2: Coronavirus related information**

1. **How would you rate your knowledge of COVID – 19 on a scale of 1 to 5?**

- 1 = no knowledge
- 2 = poor knowledge
- 3 = little knowledge
- 4 = good knowledge
- 5 = excellent knowledge

1. **Are you following the recommendations from authorities to prevent the spread of COVID-19?**

- I follow it rarely
- I try to follow it sometimes
- I follow it most of times
- I follow it at all times

1. **Have you suffered from COVID – 19?**

- Yes
- No

1. **Have anyone in your family/friends/relatives suffered from COVID – 19?**

- Yes
- No

1. **Have you ever refused or elected to forego a doctor recommended vaccine for you or someone you are responsible for (e.g. your child)?**

- Yes
- No

1. **How likely to do you think you are to get a COVID-19 vaccine when one is approved?**

- Intend to vaccinate against COVID-19
- Undecided on COVID-19 vaccination
- Do not intend to vaccinate against COVID-19
